# Pressure Measurement Techniques for Abdominal Hypertension: Conclusions from an Experimental Model

**DOI:** 10.1155/2015/278139

**Published:** 2015-05-31

**Authors:** Sascha Santosh Chopra, Stefan Wolf, Veit Rohde, Florian Baptist Freimann

**Affiliations:** ^1^Department of General, Visceral and Transplantation Surgery, Charité-University Medicine Berlin, 13353 Berlin, Germany; ^2^Department of Neurosurgery, Charité-University Medicine Berlin, 13353 Berlin, Germany; ^3^Department of Neurosurgery, University Medicine Göttingen, Georg-August University, 37099 Göttingen, Germany

## Abstract

*Introduction*. Intra-abdominal pressure (IAP) measurement is an indispensable tool for the diagnosis of abdominal hypertension. Different techniques have been described in the literature and applied in the clinical setting. *Methods*. A porcine model was created to simulate an abdominal compartment syndrome ranging from baseline IAP to 30 mmHg. Three different measurement techniques were applied, comprising telemetric piezoresistive probes at two different sites (epigastric and pelvic) for direct pressure measurement and intragastric and intravesical probes for indirect measurement. *Results*. The mean difference between the invasive IAP measurements using telemetric pressure probes and the IVP measurements was −0.58 mmHg. The bias between the invasive IAP measurements and the IGP measurements was 3.8 mmHg. Compared to the realistic results of the intraperitoneal and intravesical measurements, the intragastric data showed a strong tendency towards decreased values. The hydrostatic character of the IAP was eliminated at high-pressure levels. *Conclusion*. We conclude that intragastric pressure measurement is potentially hazardous and might lead to inaccurately low intra-abdominal pressure values. This may result in missed diagnosis of elevated abdominal pressure or even ACS. The intravesical measurements showed the most accurate values during baseline pressure and both high-pressure plateaus.

## 1. Introduction

Elevations in abdominal pressure pose a relevant risk for patients and may result in an abdominal compartment syndrome (ACS), which is associated with a high morbidity and a significant mortality [1–5]. The ACS was first described in 1989. It is defined as a sustained intra-abdominal pressure (IAP) greater than 20 mmHg combined with a new organ dysfunction [6, 7]. The pressure of 20 mmHg was standardized by the World Society of Abdominal Compartment Syndrome (WSACS) in 2006 to one of the criteria for diagnosis of ACS [8]. It is generally accepted that an early diagnosis and an aggressive therapeutic approach are crucial for the reduction of morbidity rates and overall patient survival [9]. Therefore it is necessary to have an adequate tool for correct and repetitive pressure measurements in the clinical setting. While clinical examination and radiological investigations are unreliable in the accurate diagnosis of abdominal hypertension (IAH), direct or indirect measurements of the intra-abdominal pressure (IAP) are essential [10]. Direct IAP measurement can be achieved by intra-abdominal catheters and by an intraperitoneal pressure probe and during laparoscopic surgery. Indirect methods encompass intravesical pressure (IVP), intragastric pressure (IGP), rectal, uterine, inferior vena cava, and airway pressure measurements. Based on its practicability, IAP measurement is generally performed by the intravesical approach, which is considered as the gold standard [7].

It has to be mentioned that IVP measurements are not always feasible and rely on a physiologic bladder function. Particularly in patients with bladder trauma, neurogenic bladder dysfunction, outflow obstruction, or pelvic hematomas values might be unreliable. In these patients intragastric measurements are advocated [10].

The discussion regarding the ideal location for indirect pressure measurement has not been finally resolved. In addition, there is a debate about the necessity of repeated versus single measurements for the correct diagnosis of an ACS [11]. Next to clinical experience, experimental animal studies have provided further understanding regarding improved pressure measurement techniques for the diagnosis of IAH and ACS. Based on previous studies, we created an animal model to provide experimental data on the accuracy and reliability of different measurement modalities in a broad range of intra-abdominal pressure levels. Piezoresistive probes were placed in the upper and lower abdomen for reference purposes and to register pressure differences within the intra-abdominal cavity. In addition, intragastric and intravesical measurements were obtained at different pressure levels. This was done to gain greater insight in strengths and weaknesses of different noninvasive IAP pressure measurement techniques and thus to optimize IAP measurements in clinical application.

## 2. Materials and Methods

### 2.1. Animals

Animal procedures were carried out in accordance with the international and national guidelines. The protocol was approved and supervised by the University Animal Care Committee and the federal authorities for animal research in Berlin, Germany. Six female pigs (German landrace; mean body weight of 59.5 ± 18.4 kg) were utilized for the experiments. Animals were housed 10 days prior to experiments in local stable facilities with free access to standard laboratory food and water ad libitum for acclimatization. Animals were euthanized directly subsequent to experiments under deepened anesthesia (see below) by potassium chloride injection.

### 2.2. Anesthesia and Ventilation

All surgical procedures, measurements, and euthanasia were performed under general anesthesia. Animals were deprived of food supply 12 h prior to experiments. Premedication was applied i.m. adapted to body weight with ketamine (10% 0.25 mL/kg), xylazine (0.14 2% mL/kg), azaperone (3 mg/kg), and atropine sulfate (1% 0.5 mL). General anesthesia was maintained with propofol (30 mg/kg/h i.v.) and fentanyl (3 *μ*g/kg/h i.v.). A bolus of propofol (2 mg/kg i.v.) and fentanyl (2 *μ*g/kg i.v.) was applied for euthanasia before potassium chloride injection. After endotracheal intubation, animals were ventilated in a volume-controlled mode. Positive end-expiratory pressure (PEEP) was set at 2 mmHg. End-tidal pCO_2_ was kept constantly between 42 and 48 mmHg and peripheral oxygen saturation was kept between 98 and 100%. Mean arterial blood pressure was kept during the whole procedure between 80 and 110 mmHg and measured noninvasively with an automatic sphygmomanometer (HP 66S, Hewlett Packard, Bad Homburg, Germany).

### 2.3. Invasive IAP Measurements

In order to establish direct IAP measurements, 2 custom made transdermal telemetric pressure probes based on the NEUROVENT-P-tel system (Raumedic, Helmbrechts, Germany) with a catheter length of 18 cm were implanted intraperitoneally on day 0 of experiments (IAP cranial and IAP caudal). For this purpose, 2 incisions of each 6 cm in a distance of 30 cm in the abdominal midline were made and subcutaneous pouches for the telemetric units were formed. Then the peritoneum was opened and the catheters were inserted intraperitoneally. After tight closure of the peritoneum and the fascia, the telemetric units were placed in the prepared subcutaneous pouches and the skin was carefully sutured. IAP data were read out transcutaneously by applying a radio-frequency transmission coil wired to a portable data recording device (Datalogger MPR 1, Raumedic, Helmbrechts, Germany). All measurements were stored on the Datalogger memory.

### 2.4. Noninvasive IAP Measurements

Indirect IAP measurements were performed using an intravesical pressure (IVP) measurement system (AbViser, Fa. ConvaTec, Munich, Germany) and an intragastral pressure (IGP) measurement system (CiMON, Pulsion, Munich, Germany). Both were established temporarily and directly in advance of experiments. The IVP measurement system was connected to a standard urinary catheter and referenced to the median line of the animal. The bladder was filled with 20 mL of saline before every measurement as recommended by the manufacturer. The IGP measurement system was placed together with the integrated gastric feeding tube and referenced against the atmospheric pressure. All IAP measurements were carried out at end-expiration.

### 2.5. Experimental Schedule

All relevant parameters (ventilation and blood pressure) and IAP measurements were first documented at baseline IAP in left lateral position. Then a pneumoperitoneum was induced to allow comparative IAP measurements. Therefore a Veress needle (Karl Storz, Tuttlingen, Germany) was placed intraperitoneally over a 0.5 cm skin incision directly caudal to the umbilicus. CO_2_ was insufflated until an IAP plateau of 20 mmHg was reached in a first step, and an IAP plateau of 30 mmHg was reached in a second step (Endoflator, Karl Storz, Tuttlingen, Germany). After induction of the pneumoperitoneum, measurements of parameters were carried out again 2 minutes after reaching an IAP plateau at the defined levels of 20 respective 30 mmHg given by the endoflator and repeated two minutes later. The mean value of these two repeated measurements values was taken for analysis. Animals were euthanized after decompression of the capnoperitoneum.

### 2.6. Data Analysis

The statistical environment R 2.15.2 (R Foundation, Vienna, Austria, http://www.r-project.org/) was used for data analysis. Comparison of levels of measurements between the different sources of intra-abdominal pressure was performed with the Welch *t*-tests with correction for variance heterogeneity. Imaging and calculation of the limits of agreement were performed according to the proposal by Bland and Altman [12]. A *p* value of *p* < 0.05 was considered significant.

## 3. Results

**Figure 1:**
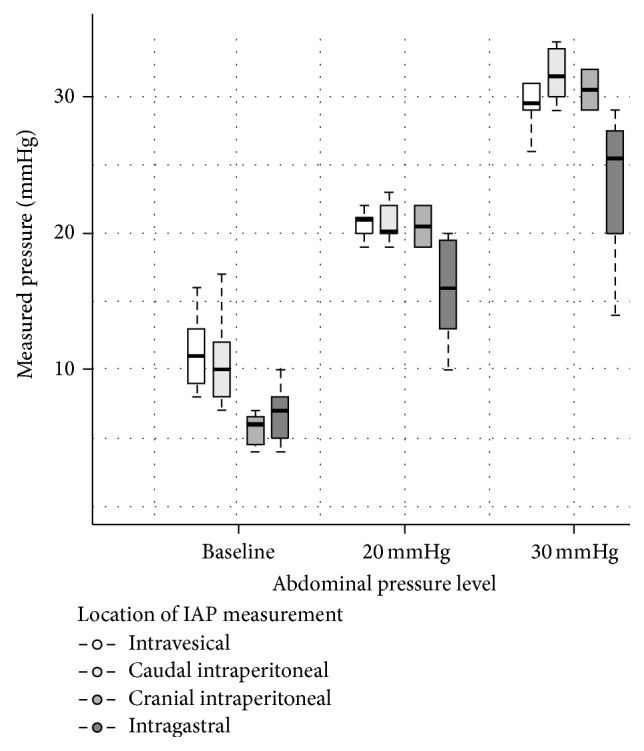
IAP values for all three different measurement techniques encompassing intravesical, invasive piezoresistive (intraperitoneal), and intragastric measurements (band = median, boxes = 25 to 75% quartile, and whiskers = 95% confidence interval). The intraperitoneal probes are subdivided after their intraperitoneal implantation site.

All operative procedures for the placement of the intra-abdominal pressure probes were carried out without any related morbidity. Pressure measurements were successfully performed in all six animals. Mean baseline IAP was 7.4 (±2.7) mmHg encompassing the cranial and the caudal invasive pressure probe measurements. The cranially implanted probes showed mean baseline IAP values of 5.6 (±1.2) mmHg, while IAP values measured with the caudally implanted probe were 9.8 (±2.4) mmHg (*p* = 0.03). After elevating the intra-abdominal pressure with the endoflator to the 20 mmHg plateau, the mean invasively measured IAP increased towards 20.7 (±1.5) mmHg. No significant differences regarding the probe position were observed at that IAP level. The cranially implanted probe showed mean IAP values of 20.5 (±2.1) mmHg, while the caudally implanted probe showed mean IAP values of 20.8 (±1.5) mmHg (*p* = 0.82). The final measurements during the 30 mmHg plateau showed a mean IAP of 31.4 (±1.9) mmHg. We noted again no significant differences of the IAP values obtained from the cranially and caudally implanted probes. The cranially implanted probe showed mean IAP values of 30.5 (±2.1) mmHg, while the caudally implanted probe showed mean IAP values of 31.6 (±1.9) mmHg (*p* = 0.49) (Figure 1).

**Figure 2:**
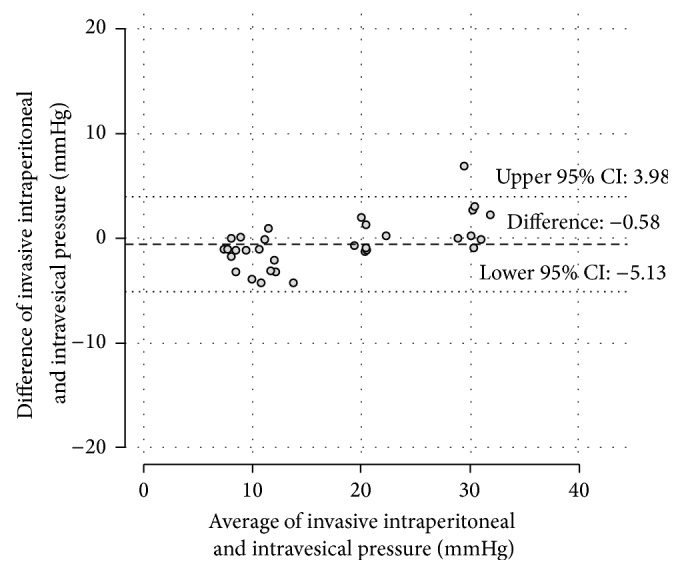
The Bland-Altman diagram plots the differences against the averages of simultaneous IAP measurements from the intravesical probes and the invasive piezoresistive probe values. No obvious signs of an increasing inaccuracy with rising values are indicated.

**Figure 3:**
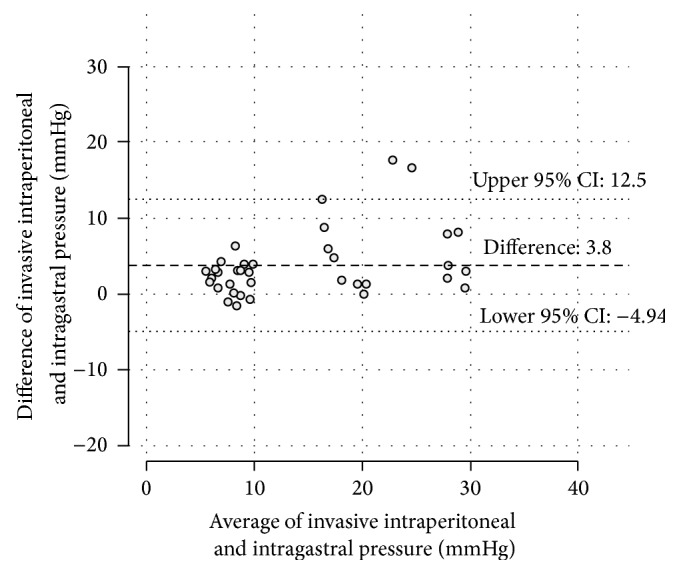
The Bland-Altman diagram plots the differences against the averages of simultaneous IAP measurements from the intragastric probes and the invasive piezoresistive probe values. An increasing divergence between the measurement modalities from the 20 mmHg plateau towards the 30 mmHg plateau is indicated.

The IGP measurements showed a mean baseline value of 6.9 (±1.6) mmHg. During the 20 mmHg plateau the mean pressure was 15.9 (±3.8) mmHg and at 30 mmHg the mean value was 23.6 (±5.6) mmHg (Figure 1). The difference from the defined pressure levels was both significant at 20 mmHg (*p* = 0.017) and 30 mmHg (*p* = 0.014). In comparison, the IVP measurements showed a mean baseline value of 9.9 (±2) mmHg. During the 20 mmHg plateau the mean pressure was 20.6 (±0.9) mmHg (*p* for difference: 0.43) and at 30 mmHg the mean value was 29.5 (±1.7) mmHg (Figure 2) (*p* for difference: 0.09). The mean difference between the invasive IAP measurements and the IVP measurements was −0.58 mmHg (*p* = 0.52). The limits of agreement, where 95% of differences between both methods are expected, were −5.13 to 3.98 mmHg (Figure 2). The bias between the invasive IAP measurements and the IGP measurements was in contrast to 3.8 mmHg, with the IVP measurements showing marginally higher values (*p* = 0.06). The limits of agreement were −4.94 to 12.5 mmHg (Figure 3).

## 4. Discussion

The abdominal compartment syndrome (ACS) is characterized by an intra-abdominal pressure (IAP) greater than 20 mmHg combined with a new organ dysfunction [13]. An IAP of more than 12 mmHg is called intra-abdominal hypertension. Different potential causes have been identified including peritonitis, pancreatitis, bowel obstruction, trauma, and forced closure of the abdominal cavity. The mortality rate of ACS is reported to be as high as 60% [2, 14]. An early and reliable diagnosis is essential for the treatment of the potentially life threatening condition of an ACS. After identifying the condition, therapeutic consequences include immediate laparotomy and abdominal decompression via temporary abdominal closure to restore organ function [15]. It has been shown that an aggressive approach based on early diagnosis and therapeutic interventions effectively decreases mortality rates.

Different methods have been applied for the measurement of intra-abdominal pressure [16, 17]. The current gold standard is the indirect measurement of the intravesical pressure. Although direct measurement probes produce more accurate values their clinical implementation is restricted due to their invasive character [18].

IVP measurements are not feasible in some patients for a various set of reasons [10]. Intragastric measurements are generally advocated in this patient population.

We created a large animal model to simulate an abdominal compartment syndrome ranging from 20 to 30 mmHg. Three different measurement techniques were applied, including telemetric piezoresistive probes for direct pressure measurement and intragastric and intravesical probes for indirect and noninvasive measurements. All three techniques were suitable to detect elevated abdominal pressures, although significant differences could be obtained.

The mean baseline values of the IVP measurement and the caudal intraperitoneal probe were almost identical (9.8 versus 9.9 mmHg). The cranial intraperitoneal probe showed lower values (5.6 mmHg). These significant differences between the cranial and the caudal intraperitoneal probes during baseline measurement might be explained by their different anatomical positions and the actual hydrostatic pressure at their site [17]. The previously seen differences between both intraperitoneal probes vanished during both high-pressure plateaus. In our hypothesis is the hydrostatic pressure effect eliminated at high-pressure levels, which leads to the almost identical values for the cranial and caudal probes.

The piezoresistive intraperitoneal probes (cranial and caudal probe combined) and the intravesical probes showed the most accurate values during both high-pressure plateaus. The intragastric data showed a strong tendency towards false low values. We measured mean values of 15.9 mmHg at the 20 mmHg plateau and 23.6 mmHg at the 30 mmHg plateau. The maximum deviation was 16 mmHg in one animal. The limits of agreement were clinically unacceptably high. Furthermore, a systematical underestimation of the IAP measured can occur in clinical practice due to a standard head of bed elevation, which causes a vertical pressure difference between the stomach and the bladder.

Intragastric IAP measurements are on the basis of our data not suitable for the detection of ACS. Nevertheless, this is an experimental animal study with small group size, and its clinical applicability is therefore limited. Further clinical investigation is warranted.

## 5. Conclusion

We conclude that the intragastric pressure measurement is potentially hazardous leading to inaccurately low values and may result in underdiagnosed elevated abdominal pressure or even ACS.
